# Synthetic versus autologous reconstruction (Syn-VAR) of the medial patellofemoral ligament: a study protocol for a randomised controlled trial

**DOI:** 10.1186/s13063-018-2622-7

**Published:** 2018-05-03

**Authors:** Adam Tucker, Sam McMahon, Bronwyn McArdle, Bridgeen Rutherford, Danny Acton

**Affiliations:** 1 Altnagelvin Area Hospital, Glenshane Road, Londonderry, BT47 6SB Northern Ireland; 2 R+D Office, Clinical Translational Research and Innovation Centre (C TRIC), Altnagelvin Area Hospital, Glenshane Road, Londonderry, BT47 6SB Northern Ireland

**Keywords:** Knee, Medial patellofemoral ligament, MPFL, Hamstring, Reconstruction, Randomized control trial (study type), Outcomes

## Abstract

**Background:**

Recurrent patellar instability incidence is 5.8/100,000 population, and recurrent dislocations are reported in the range of 15–80%. Recurrent instability is multifactorial and can be associated with disorder of limb alignment, osseous development, congruity of the patella in the trochlea and soft tissue static and dynamic constraints. The multifactorial aetiology makes management challenging, and a lack studies in a heterogeneous population with robust clinical outcomes compounds this further. The options for medial patellofemoral ligament (MPFL) reconstruction include autologous graft reconstruction with semitendinosus tendon, or synthetic polyester woven grafts. In theory, in the young active patient, the surgeon may wish to preserve the hamstring tendons to reduce postoperative morbidity to the patient, reduce delay in recovery from donor site morbidity and preserve the hamstring tendons. There have been no randomised controlled trials (RCTs) to date that directly compare autologous hamstring and synthetic reconstruction methods. This trial aims to assess the functional outcomes in those undergoing MPFL reconstruction with either autologous hamstring graft reconstruction, or a commercially available synthetic polyester open woven tape.

**Methods:**

Following a power calculation using previous studies as the pilot data, a total of 30 patients will be included in the study. Enrolment is based upon strict inclusion/exclusion criteria outlined in the “Methods”. Participants will be randomized to receive either autograft or synthetic graft reconstruction. We aim to recruit 15 patients to each arm of the study. Surgery is performed by a single consultant surgeon experienced in both reconstructive options, using the default surgical technique for each. A postoperative physiotherapist-directed rehabilitation protocol will be implemented, as is routine. The primary outcome is the Kujala functional score and its change over the study period. Data on further secondary outcomes using validated outcomes scores will also be collected, specifically the Tegner and Lysholm, Banff Patellar Instability Index, and ACL Quality of Life Score. Secondary outcomes are complications and revision for any reason. The patient follow-up time is 2 years. The first patient will be recruited in January 2018. The expected trial deadline for recruitment is December 2018, with records and results being held for 5 years.

**Discussion:**

This RCT study is the first to directly compare the efficacy of autograft versus synthetic allograft in MPFL reconstruction and the graft effects on patient-reported clinical outcomes.

**Trial registration:**

ISRCTN, 16657952. Registered on 3 March 2017.

The study protocol has been approved by the Office for Research Ethics Committees of Northern Ireland (ORECNI 17/NI/0129).

**Electronic supplementary material:**

The online version of this article (10.1186/s13063-018-2622-7) contains supplementary material, which is available to authorized users.

## Background

Recurrent patellar instability incidence is 5.8/100,000 population, and reported recurrent dislocations are in the range of 15–80% [1, 2], with recurrence >50% after two dislocations [3]. Up to 55% of patients are unable to return to their pre-injury level of activity [4]. This can be particularly difficult to manage due to the multifactorial nature of patellar instability [3, 5], and a myriad of surgical options are described [6]. These include imbrication [7], repair by both open [8, 9], and arthroscopic techniques [10, 11] and reconstruction using a variety of graft options [9, 12–16].

The medial patellofemoral ligament (MPFL) is the most important restraint to lateral displacement of the patella from 0° to 30° of flexion, providing up to 60% of lateral patellofemoral stability [17]. MPFL insufficiency is present in 90% of acute, and 100% of recurrent, patellar dislocations [18]. Outcomes following MPFL reconstruction with autograft are reported as good to excellent, with increases in validated outcome measures for patellar instability, namely the Kujala score [3, 19–22].

Synthetic graft outcomes are also encouraging with studies reporting increases in Kujala outcome scores and patient-reported success rates of up to 94% [23–27]. One study reports the outcomes as being comparable to those of autograft reconstruction, with minimal need for revision intervention [25]. Stability is further enhanced when MPRL reconstruction is performed in combination with vastus medialis advancement compared to MPFL reconstruction alone [27].

Potential complications of MPFL reconstruction include re-dislocation, postoperative quadriceps dysfunction, adverse reaction to synthetic graft material, persistent pain and patellar fracture, but these risks are low and quoted as 5–10% [28–30]. Whilst multiple options are described, there is a paucity of clinical comparative studies. No study has compared autologous to synthetic graft reconstruction.

The objective of the current paper is to present a randomised controlled trial designed to investigate the difference, if any, between autologous semitendinosus synthetic graft MFPL reconstruction in patients with patellar instability.

## Methods

### Trial design

A single-centre, non-blinded, randomised control trial will be performed in Altnagelvin Area Hospital, Northern Ireland. This is a district general hospital with trauma and orthopaedic capabilities, serving the north and west of the province – a population of approximately 400,000 individuals.

The study aims to investigate the functional outcomes following MPFL reconstruction using either autologous hamstring reconstruction, or a synthetic open weave polyester tape (NeoLigament, Leeds, UK). This trial is not considered as a new interventional assessment, as both techniques are widely accepted and reported in the literature individually. However, no randomised controlled study has been performed evaluating these techniques head to head.

The study protocol (version 2) is registered with the ISRCTN (ISRCTN 16657952, March 2017), and contains the information required by the World Health Organisation Trial Registration Data Set.

Regional ethical approval was granted by the Office for Research Ethics Committees (REC) of Northern Ireland (ORECNI ID 17/NI/0129) on 21 August 2017.

**Fig. 1:**
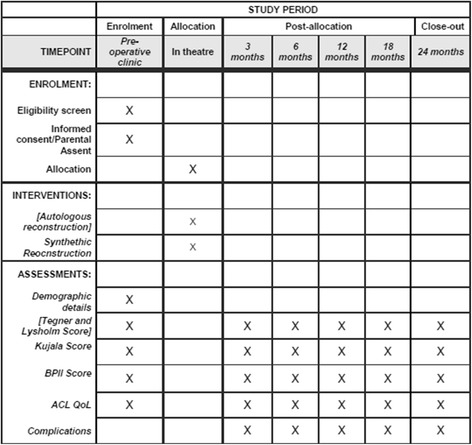
Example template of recommended content for the schedule of enrolment, interventions, and assessments. BPII, Panff Patellar Instability Index; ACL QoL, Anterior Cruciate Ligament Quality of Life Score

The trial was designed using the Recommendations for Interventional Clinical Trials (SPIRIT) 2013 statement (Additional file 1). Figure 1 shows an example template of recommended content for the study. The Coleman Methodology Score (CMS) was used to assist with the trial design, ensuring validity and reliability of the study, and accordingly to allow for future meta-analyses [31]. The current CMS of the trial design is 79/100, equating to a rating of “good” (good 70–84, excellent 85+).

Collected data will be kept in the hospital notes in a designated study sub-section. All data will also be held on a secure hospital-server shared drive, accessible only to those registered as trial investigators. Patients will be designated alphanumeric codes rather than be identified by personal details. Data will be entered by either the Principal Investigator or a member of the trial team, then checked and verified by one of the additional collaborating authors. Data monitoring will be performed by the study unit Research and Development (R&D) team and/or the Regional Ethics Committee at their discretion.

Changes in trial design will be conveyed to the study unit R&D Department and the REC for ethical review. Any changes to the trial protocol will also be reflected in the ISRCTN documentation.

The data analysis will be by an intention-to-treat protocol, which will minimize bias between the treatment arms, and demonstrate the efficacy of the intervention itself.

### Participants

Patient enrolment will begin in November 2017. Patients with patellar instability due to rupture of the MPFL are seen at routine clinics at the study institution. Patients presenting with suspected MPFL-related instability will be streamlined to attend the Principal Investigator’s clinic to assess suitability for inclusion.

Those deemed eligible for inclusion will have completed a comprehensive physiotherapy rehabilitation programme. The standardized protocol for conservative therapy is outlined in Additional file 2. All patients will have up-to-date routine radiographs to include anteroposterior, lateral and skyline views of the affected knee, plus magnetic resonance imaging (MRI) of the knee (or computed tomography (CT) if MRI is contraindicated). Indications for surgery will include ongoing symptomatic instability, clinical evidence of MPFL laxity, or ligament signal attenuation on MRI.

**Fig. 2:**
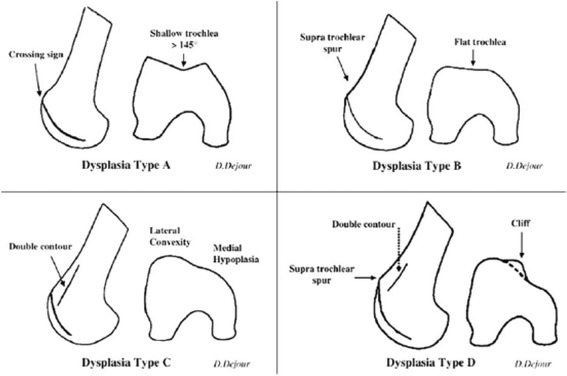
The Dejour classification of trochlear dysplasia as proposed by Dejour and Le Coultre [42]. Type A – crossing sign on lateral radiograph, shallow trochlea > 145°. Type B – supratrochlear spur, flat or convex trochlea. Type C – double contour sign on lateral radiograph, hypoplastic medial femoral condyle. Type D – supratrochlear spur, double contour sign and cliff pattern between the femoral condyles

**Fig. 3:**
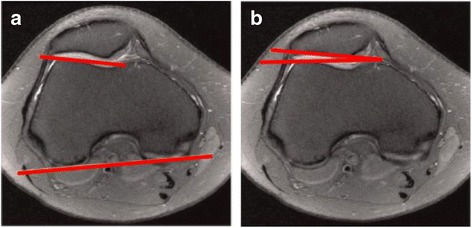
Method for measuring patellar tilt. Fulkersons angle (**a**) is the angle of the lateral facet of the patella, relative to the line connecting the posterior condyles on axial computed tomography/magnetic resonance imaging. Angle of Laurin (**b**) – the lateral patellofemoral angle assessed with the knee in 20° of flexion. This angle is formed by the lateral patellar facet and a line drawn across the most prominent aspect of the anterior portion of femoral trochlea

Inclusion and exclusion criteria are defined in Table 1. Trochlear dysplasia will be classified according to the Dejour classification (Fig. 2). Patellar tilt will be defined by measuring Laurin and Fulkerson’s angle (Fig. 3).

**Table 1:** Inclusion and exclusion criteria

Inclusion criteria	Exclusion criteria
Age 14 years or older	Abnormal hip or hind foot pathology
Closed proximal tibial physes	Excessive femoral anteversion (> 30 degrees) and/or excessive tibial torsion (> 40 degrees)
Symptomatic patella instability	Excessive coronal plane deformity of the knee (> 10 degrees)
Positive clinical findings	Previous surgical stabilisation
Two or more dislocations	Evidence of lateral compression on skyline view
Failed to respond during a reasonable period (> 3 months) of conservative management and physiotherapist-directed rehabilitation protocol	Dejour grade C/D trochlea
	Excessive patellar tilt
	Open tibial physes
	CT/MRI confirms presence of secondary arthritic changes
	Patients who require bony correctional procedures to restore alignment
	Known hypersensitivity to synthetic graft material
	Previous peri-articular infection on affected side
	Presence of osteochondral lesions on MRI/CT requiring surgical intervention
	Positive findings of a multiligamentous knee injury

*CT* computed tomography, *MRI* magnetic resonance imaging

Standard radiological indices of patellar height will be measured using accepted, standardized methods (Insall Salvati and Caton-Deschamp indices). MRI/CT will be used to assess the tibial tuberosity to trochlear groove (TT-TG) distance and identify any additional pathology within the affected knee. Patients will be excluded if bony realignment procedures are required to restore patellar stability. This will be determined by a TT-TG > 20 mm on CT/MRI, or a Dejour tpe C/D trochlea.

### Intervention

The intervention will be reconstruction of the MPFL using either autologous gracilis or semitendinosus tendon, or synthetic open weave polyester tape (Neoligament, Leeds UK), with advancement of the vastus medius obliquitus (VMO). All surgery will be performed by the Principal Investigator, who is a consultant with significant specialist interest and expertise in soft tissue knee reconstruction, and who has performed significant numbers of both techniques. This will eliminate any learning curve between techniques. All patients will be reviewed preoperatively the by Principal Investigator to ensure eligibility for inclusion. Consent will be gained by the Principal Investigator or a suitably trained orthopaedic registrar, who is part of the clinical trial team.

Patients will undergo general anaesthetic for surgery. Routine intravenous antibiotic prophylaxis will be administered in accordance with local Trust policy. Patients will be positioned in a supine position with a high thigh-tourniquet. A side support is used to support the leg in 90° of flexion. Diagnostic arthroscopy will not be performed as all patients with evidence of an osteochondral lesion on preoperative MRI will be excluded from the study. Reconstruction using either a hamstring autograft or synthetic graft will be performed as per the surgeon’s default technique. Should there be a breach of the synovial lining, this will be repaired to ensure a purely extra-articular reconstruction is performed.

The autograft tendon will be harvested through an approximate 3-cm vertical incision over the pes anserinus with a vertical opening to the bursa and exposure and release of the tendon using a tendon stripper. The senior author’s preference is to use the gracilis tendon, unless it is of a poor quality, or if the patient body habitus requires a more substantial graft, then the semitendinosus tendon will be harvested instead.

An approximately 6-cm straight medial incision will be made over the distal femur, two thirds of the distance between medial epicondyle and the patella. Extra-articular dissection to the medial patellofemoral retinaculum, with identification of the medial border of the patella, will be performed. With the knee flexed to 90°, the 3.2-mm drill is passed from the medial edge of patella through to the midline of the proximal pole of the patella. The graft is passed through the patella, and then brought back medially underneath the quadriceps tendon under the VMO. This will create a triangular shape for the completed reconstruction.

The medial aspect of the distal femur is identified and prepared at a point half way between the adductor tubercle and the medial epicondyle. This is the location of the isometric point (often referred to as Schottel’s point), which is approximately 45° to the long axis of the femur. Identification of the isometric point in flexion and extension on the femur can then be judged by the patella being correctly positioned in the trochlear groove throughout the full range of movement. Care is taken to avoid excessive tension on the graft, particularly in extension.

For autologous graft reconstruction, a Beath pin is drilled in from the isometric point in a medial-to-lateral direction. A 6–7 mm cannulated drill bit is then used to ream over the Beath pin. After the graft has been looped through the patella, it can then be passed through the tunnel and secured using a 7 mm × 25 mm interference screw.

For synthetic reconstruction, the isometric point serves as a landmark for the insertion of the Fastlok device to anchor the synthetic graft, whilst minimizing metalwork prominence. The poly-tape can be threaded onto the Fast-Lok device and inserted to the isometric point using the impactor, buried into the distal femur, and then cutting the loose ends with a short tail.

The VMO fascia is then incised 2 cm proximal to the terminal muscle belly with a curvilinear incision. The tendon is left intact whilst the muscle belly is raised, advanced and used to cover any exposed poly tape remnants and to encourage fibrous ingrowth of the tape. A double-breasted repair with absorbable 2/0 Vicryl will be performed.

Wound closure using a 2/0 Vicryl subcutaneous continuous stitch, followed by a subcuticular 3/0 Vicryl Rapide will be peformed. Local anaesthetic, usually 0.5% Chirocaine, is infiltrated into the wounds, before application of dressings, wool and crepe. A knee extension brace will be applied in the immediate postoperative period.

### Postoperative rehabilitation

Rehabilitation will be supervised by a trained physiotherapist using the study unit default MPFL rehabilitation protocol. Initially, the patient’s knee will be placed into a hinged knee brace set at 0–30° of flexion for 2 weeks. Ankle isotonic exercises using graded elastic resistance bands will be provided, along with isometric quadriceps, gluteal and hamstring exercises for all patients. Further closed chain quadriceps exercises will also be undertaken. Knee flexion beyond 90° will be avoided postoperatively during the initial 6 weeks.

At 1–2 weeks after surgery, quadriceps activation exercises in the form of static quadriceps exercises, straight leg raises and inner quadriceps rehabilitation will be performed. Hip abductor strengthening will also begin.

From weeks 2–4 after surgery, gait optimisation with an active range of motion from 0° to 60° of flexion shall be permitted. Quadriceps activation exercises will continue, with additional electromuscular stimulation (EMS) if the VMO recruitment is inadequate. Further exercises as per weeks 1–2 will continue.

During weeks 4–6, the patient will be allowed 0–90° of flexion and full weight-bearing will be promoted. Further strengthening of quadriceps and hip abductors will continue. Proprioceptive rehabilitation will begin at this stage also, in combination with core strengthening and flexibility exercises targeting the quadriceps, hamstring and calf muscles.

During weeks 6–12 the rehabilitation thus far will be further developed, with the aim of progression to achieving good control with short functional arc movements. Weight transfer to the leg that has undergone surgery will commence, with a focus on achieving single-leg standing control. Additional supervised weights-based strengthening of the hips and knee can commence at this stage. The aim is to achieve a pain-free, full range of motion on the operative side, which allows independent mobility (i.e. dynamic stability and eccentric control on single-leg stance).

From 12 weeks to 6 months, the aim will be to achieve Medical Research Council (MRC) grade 5 muscle strength in the leg that has undergone surgery. EMS may be used as an ongoing adjunct if required at this stage. If the patients can perform a controlled single-leg squat at this time, then plyometric rehabilitation can begin with hopping and change of direction exercises. Further proprioceptive rehabilitation with wobble boards, trampettes and dyna-cushions will begin. Light jogging may commence provided the knee is dynamic stable. The full rehabilitation protocol is included in Additional file 3.

### Outcomes

The primary outcome will be to determine the patient-reported outcome score using the validated Kujala score [32]. This is a validated scoring system designed specifically for patellar instability [32]. Scores will be collated preoperatively, and at the pre-defined postoperative time points of 3, 6, 12, 18 and 24 months. This will be identical for both autologous and synthetic reconstruction techniques. Specifically, we will report the change in the Kujala score at these set time points and analyse the data for differences between surgical techniques to determine if one appears to be superior. This will demonstrate the efficacy of the treatments under investigation, and allow a meaningful statistical comparison between the treatment arms.

Additional secondary outcomes scores will also be reported. These will be collected at the same time points as the primary outcome score, preoperatively and then postoperatively at 3, 6, 12, 18 and 24 months. The outcome scores will include the Tegner Activity Level Scale [33], Lysholm Knee Score [34], the Banff Patellar Instability Index (BPII) [35] and the anterior cruciate ligament (ACL) Quality of Life Score [36]. These scores have been previously validated and are reliable in assessing ligamentous injuries of the knee and are commonly reported in studies of ligamentous reconstruction.

Other secondary outcomes will include the incidence of complications and all-reason revision surgery. Those patients lost to follow up will have their data included up to this point to allow for analysis at the set postoperative time points. The aim is to have data on medium-term outcomes at 2 years for all participants.

At each study-review time point, participants will be questioned about adverse events attributable to undergoing surgical reconstruction of the MPFL that they have experienced since their previous review. This will include graft rupture/failure, synovitis, persistent instability and reoperation/revision surgery.

### Sample size

Sample size was calculated using a web-based calculator (www.openepi.com). Results from a previous study using an ACL autologous reconstruction technique were used to define the control group [21]. The authors reported a postoperative increase in the Kujala score from 56.7 ± 17.7 (2×SD) to 86.8 ± 14.4 (2×SD). Success rates of isolated MPFL reconstruction are good, with patients reporting good or excellent outcomes of 87–96% [3, 21].

The minimally clinically important difference is defined as half of a standard deviation according to the method utilized by Norman et al*.* [37]. Using the result from Panni et al., we determined that σ = 7.2 in the treatment cohort, and therefore assumed a difference in 10 points would be clinically detectable and relevant.

Using a 1:1 enrolment ratio in a non-inferiority trial design, with beta of 0.2 and alpha of 0.05, a total of 30 patients will be required – 15 in each treatment arm. A Cohen’s *d* value of 1.01 with an effect size of 0.58 means that any significant difference attributable to the treatment received should be found using this sample size. Using the results of a previously published study [21], a minimum sample size of 10 patients – 5 in each arm – will be required in order to detect a clinical difference in the treatment groups.

Therefore, the aim is to recruit 15 patients in each group into this study, which allows for an attrition rate of up to 60% in each group. This will still allow meaningful statistical analysis to be performed. This is relevant due to the young age of the study population, which therefore has potential to be affected by population migration and loss to follow up. Clinical follow up will occur postoperatively at intervals of 3, 6, 12, 18 and 24 months.

### Randomisation and blinding

The allocation to the surgical technique to be used will be placed in an opaque, sealed envelope by a study investigator. Once sealed, the envelopes will be randomly mixed before being numbered consecutively. No pre-stratification is required. The Principal Investigator will be blind to this process throughout.

Once the patient has arrived in the anaesthetic room of the operating theatre, the randomisation envelopes will be drawn in consecutive numerical fashion by the Principal Investigator and a member of the theatre team. This will determine which surgical technique the patient will receive. A master file containing a log of those involved in randomisation will be used for verification purposes.

Patients will be blinded to their study group at the time of entering theatre. It is not possible to blind the operating surgeon/Principal Investigator. No “sham” incisions will be used to blind the patients undergoing surgery to the intervention allocated. Unblinding will be permissible should patients present with a postoperative complication that requires repeat surgical intervention.

### Statistical analysis and trial end points

The intended data analysis will be on an intention-to-treat and per-protocol basis to demonstrate non-inferiority. This will allow bias between the treatment arms to be minimised, and demonstrate the efficacy of the intervention itself. Data analysis will be performed by one of the study investigators using SPSS v22 for Mac (IBM Inc., Armonk NY, USA), and will be verified by the local unit R&D department. Data will be tested for normality using the Shapiro Wilk test. Appropriate parametric and non-parametric tests will used accordingly.

Primary outcome measures will be compared between groups at set postoperative time points of 3, 6, 12, 18 and 24 months. Additionally, outcomes at these time points will be compared to preoperative baseline i.e. the analysis metric will be the change from preoperative baseline. This will allow calculation of change in patient-reported outcome scores at the set time points and the change in these reported scores over the study timeframe.

For primary and secondary outcome scores, the mean ± SD, median and range will be given for continuous data. For the secondary outcomes, the frequency of complications and all-reason revision will be recorded as a percentage and analysed by chi square analysis. For all tests, a *p* value <0.05 will be considered significant.

End points will be completion of the study timeframe (2 years), or further dislocation/all reason revision surgery. The trial will only be stopped should information become available that indicates that it is unethical to use synthetic grafts.

We propose to test non-inferiority using the intention-to-treat analysis set. No crossover of treatment arms will be allowed to occur as part of the protocol. Therefore, the study shall include all participants, as randomised. Patients will be reviewed at each specified time point as previously outlined. A single imputation model with last value carried over (LVCO) will be employed should patients drop out or be lost to follow up. The trial is designed to accommodate a 60% dropout rate, and still maintain a suitable sample size for meaningful statistical analysis.

### Recruitment

Patients will be recruited at Altnagelvin Area Hospital, Northern Ireland, having met the inclusion criteria and been fully informed of the study in the outpatient clinical setting. Within 14 days of surgery, patients will be reviewed and will be fully informed of the programme, and consent will be obtained. If the patient consents to participate in the study, they will undergo randomisation by the coordinating investigator as previously described. Patients may withdraw themselves at any time during the study; however, data up to this point will be included in any statistical analysis.

## Discussion

Patellar instability conveys significant morbidity to the young active population, which is further impacted in the case of recurrent instability [1–3]. There are multiple causes of patellar instability, some which require isolated soft tissue reconstruction, and others that require bony realignment surgery. Attempts to rationalize treatment with algorithmic evidence-based decision matrices have been published [3]; however, the multifactorial nature of the condition predisposes to confounding factors [17]. Using this information, we have developed the current trial in an attempt to create a homogenous population in order to assess two treatment options and minimise any confounding factors.

Previous studies have demonstrated appropriate outcomes using autologous hamstring and synthetic tape reconstruction. However, the majority of literature pertaining to these is of low quality, lacking in homogenicity, based on retrospective design and have potential recall and publication bias, as most are from high-volume centres [17, 38].

Berruto reported good increases in Kujala knee scores at 3 years following reconstruction using a bioactive synthetic ligament for MPFL reconstruction in 16 patients [26]. The authors also demonstrated a reduction in visual analogue scale pain scores compared to preoperative scores and an overall satisfaction rate of 88%. Risk of revision was low and this was for medial epicondylar pain.

Nomura and colleagues have extensive reported on the use of artificial ligaments for MPFL reconstruction. They have shown good outcomes in 27 knee joints, at a midterm follow up of 5.9 years, with a 96% satisfaction rate [25]. Furthermore, they have studied histological specimens and found that “ligamentization” occurs over time in the synthetic ligaments [39, 40]. In another study with a mean follow up of 11.9 years, an 88% success rate was reported with significant increases in the Kujala score from baseline, with minimal evidence of osteoarthritis in the long term [41].

This is the first study to directly compare autologous and synthetic grafts for MPFL reconstruction. The use of validated outcome scores, combined with a rigid study design, should allow definition of the indications for isolated MPFL reconstruction. Besides providing information on the reduction in pain, improvement in symptoms, the restoration of daily activities and the incidence of complication rates for MPFL reconstruction, the study will permit surgeons to fully inform patients requiring this intervention.

We present the rationale and design of a pragmatic randomised controlled trial in which patients are blinded to intervention allocation, comparing the use of autologous hamstring to synthetic ligament reconstruction, in patients with recurrent patellar instability due to confirmed disruption of the MPFL. The results of this trial will be beneficial for orthopaedic surgeons in defining the criteria for performing this surgery, and enable them to advise the optimal treatment choice for MPFL reconstruction. The first study results are expected in 2020 and will be communicated via publication and presentation at national and international meetings.

### Trial status

Patient recruitment and intervention began in January 2018.

## Additional files

Additional file 1: SPIRIT 2013 checklist: recommended items to address in a clinical trial protocol and related documents. (DOC 120 kb)

Additional file 2: MPFL reconstruction postoperative rehabilitation protocol. (DOCX 22 kb)

Additional file 3: Rehabilitation guidelines for patients with patella dislocation. (DOCX 15 kb)

## Abbreviations

ACL: Anterior cruciate ligament
BPII: Panff Patellar Instability Index
CMS: Coleman Methodology Score
CT: Computed tomography
EMS: Electromuscular stimulation
ISRCTN: International Standard Randomised Controlled Trials Number
MPFL: Medial patellofemoral ligament
MRC: Medical Research Council
MRI: Magnetic resonance imaging
ORECNI: Office for Research Ethics Committees of Northern Ireland
R&D: Research and development
RCT: Randomised controlled trial
REC: Research Ethics Committee
SPIRIT: Standard Protocol Items: Recommendations for Interventional Trials
SPSS: Statistical Package for the Social Sciences
VMO: Vastus medius obliquitus
