# Supplementary material for: Synthetic versus autologous reconstruction (Syn-VAR) of the medial patellofemoral ligament: a study protocol for a randomised controlled trial
Source: Trials. 2018 May 3;19:268. doi: 10.1186/s13063-018-2622-7 (PMC5934878; doi:10.1186/s13063-018-2622-7)
Supplement: Supplementary file 2 — MPFL reconstruction postoperative rehabilitation protocol. (DOCX 22 kb) [file 13063_2018_2622_MOESM2_ESM.docx]

**MPFL Reconstruction Post-Operative Rehab Protocol**

Rehabilitation will be supervised by a trained physiotherapist using the study unit’s default MPFL rehabilitation protocol.

Initially, the patient will be placed into a hinged knee brace set at 0-30degrees flexion for 2 weeks. Ankle isotonic exercises using graded elastic resistance bands will be provided, along with isometric quadriceps, gluteal and hamstring exercises for all patients. Further closed chain exercises for the quadriceps will be undertaken also. Knee flexion beyond 90 degrees will be avoided during the initial 6 weeks post-operatively.

**On discharge from ward patient should be:**

- Independent and safe mobilizing with appropriate walking aid/s, including transfers and stairs if necessary.
- Independent and safe with home exercise program and brace management.
- Achieving full range of knee extension and 30° of knee flexion.
- Referred for ongoing out-patient physiotherapy with an appointment arranged for within 2 weeks post-operatively

**Phase 1: week 1-2**

At 1-2 weeks post operatively, quadriceps activation exercises in the form of static quadriceps exercises, straight leg raises and inner quadriceps rehabilitation will be performed. Hip abductor strengthening will also begin.

**Goals:**

- Achieve full range of knee extension and 30° of knee flexion
- Achieve quads activation / VMO activation / SQ / IRQ / SLR
- Hip strengthening exercises with theraband for abduction and adduction

Sample exercises: Active warm-up, ROM ex’s

Flexibility: hamstrings, gastroc-soleus complex, hip flexor

Strength: quads sets, SLR 4-ways, TKE against theraband

**Criteria for progression to next Phase:**

- No signs of active inflammation
- Patient is able to establish a good volitional quads set in TKE
- Patient can achieve 30° flexion

**Phase 2: week 2-4**

From weeks 2-4 post operatively, gait optimization with an active range of motion from 0-60 degrees of flexion shall being. Quadriceps activation exercises will continue, with additional electromuscle stimulation (EMS) if the VMO recruitment is inadequate. Further exercises as per weeks 1-2 will continue.

**Goals:**

- Achieve full range of knee extension and 60° of knee flexion
- Achieve quads activation / VMO activation / SQ / IRQ / SLR, CKC ex’s only
- Hip strengthening exercises with theraband for abduction and adduction

Sample exercises: Active warm-up, ROM ex’s

Flexibility: hamstrings, gastroc-soleus complex, hip flexor

Strength: quads sets, SLR 4-ways, TKE against theraband, EMS as necessary

Gait training, balance & proprioception

**Criteria for progression to next Phase:**

- No signs of active inflammation, minimal joint effuson
- Patient is able to establish a good volitional quads set in TKE
- Patient can achieve 60° flexion

**Phase 3: week 4-6**

Week 4-6 will allow 0-90degrees of flexion, and full weight bearing will be promoted. Further strengthening of quadriceps and hip abductors will continue. Proprioceptive rehabilitation will begin at this stage also, in combination with core strengthening and flexibility exercises targeting quadriceps, hamstring and calf muscle

**Goals:**

- Achieve full range of knee extension and 90° of knee flexion
- Achieve full quads activation, ISLR
- FWB with mobility aid/s

Sample exercises: Active warm-up, ¼- ½ revolutions on bicycle (per ROM restrictions)

Flexibility: hamstrings, gastroc-soleus complex, hip flexor, ITB

Strength: quads sets, SLR 4-ways, TKE against theraband, wall slides,

Hamstrings isotonics, heel raises, bridging +/- core work

Gait training, treadmill walking program

**Criteria for progression to next Phase:**

- No signs of active inflammation
- Patient is able to establish an independent SLR
- Patient can achieve 90° flexion
- Patient is FWB with walking aid/s
- Patient has regained flexibility of hamstrings, gastroc-soleus complex, hip flexor & ITB

**Phase 4: week 6-12**

Weeks 6-12 will develop upon the rehabilitation so far, with progression to achieving good control with short functional arc movements. Weight transfer to the operated leg will commence, with a focus on achieving single leg standing control. Additional supervised weights based strengthening of the hips and knee can commence at this stage. The aim is achieve a pain free, full range of motion on the operative side, which allows independent mobility (i.e dynamic stability and eccentric control on single leg stance).

**Goals:**

- Progress towards full flexion ROM
- FWB wean off walking aid/s
- Progress towards unsupported single leg stance by 12 weeks
- Progress towards unsupported step down by 12 weeks

Sample exercises: Active warm up : bike, stepper

Flexibility: hamstrings, gastroc-soleus complex, hip flexor, ITB

Strength: quads sets, SLR 4-ways, TKE against theraband, heel raises,

Step-ups/downs, lunges, squats, leg press

Balance/Proprioception: Progress double limb to single limb

Gait: Initiate treadmill walking

**Criteria for progression to next Phase:**

- Full ROM at knee
- Normal gait pattern FWB without walking aid/s
- Good PFJ mobility & control (no lateral tracking or signs & symptoms of instability)
- Patient can perform unsupported single leg stance
- Patient can perform unsupported step down
- All exercises are pain free

**Phase 5: From 12 weeks to 6 months**

The aim will be to achieve MRC grade 5 muscle strength in the operated leg. EMS may be used as an ongoing adjunct if required at this stage. If the patients can perform a controlled single leg squat at this time, then plyometric rehabilitation can begin with hopping and change of direction exercises. Further proprioceptive rehabilitation with wobble boards, trampettes and dyna-cushions will begin. Light jogging may commence provided dynamic stability is present.

**Goals:**

- Achieve controlled full ROM of knee
- Non-antalgic gait pattern, no walking aid/s
- Exercises should be pain free
- Able to dynamically stabilize knee with good eccentric control during single leg stand/squat
- Unsupported single leg squat before introduction of plyometrics
- Return to gym-based activities & sport specific activities
- Single leg press should be equal to or greater than non-operated leg
- Anticipate return to sport at 6 months (though may take longer)

Sample exercises: ROM, strengthen through range

Functional dynamic work including hopping & jumping

Gradual return to plyometric exercises +/- height, +/- cushions

Sport specific & occupation specific rehab

Initiate treadmill jogging program

**Criteria for progression to next Phase:**

- All exercises are pain free
- Controlled full ROM of knee
- Full strength MRC grade 5 of operated leg
- Patient can perform a single leg hop test at 85% of non-operated
- No signs or symptoms of patellar instability
- Normal gait mechanics

**Criteria for progression to sport:**

- Patient has no exacerbation of symptoms progressing to functional sport program
- No effusion
- Full ROM
- Patient can demonstrate confidence, using patient reported outcome measure, Tegner-Lysholm knee score (‘’excellent’’ >90)
- Quads strength should be within 90% of uninvolved side
- Patient can demonstrate functional stability: Hop tests should be within 85-90% of the uninvolved side before return to sport (testing can guide treatment, assessing for bilateral asymmetries).

At each review time point (pre-op, 3/6/12/18/24 months post op), quadriceps leg strength will be compared for both the operative and non-operative leg suing a Functional Squat Machine (FSM) to monitor progress and compare rate of rehabilitation between synthetic and autologous grafts.

**Failure to progress**

**Swelling, Pain, Breakdown of wound, Recurrent instability, Numbness / altered sensation**

These will be raised and discussed with the PI, and clinical review may be brought forward in this instance to avoid issues. Provided that none of these issues result in patients meeting the end points defined in the study protocol, they will continue in the trial.
