# Supplementary material for: Synthetic versus autologous reconstruction (Syn-VAR) of the medial patellofemoral ligament: a study protocol for a randomised controlled trial
Source: Trials. 2018 May 3;19:268. doi: 10.1186/s13063-018-2622-7 (PMC5934878; doi:10.1186/s13063-018-2622-7)
Supplement: Supplementary file 3 — Rehabilitation guidelines for patients with patella dislocation. (DOCX 15 kb) [file 13063_2018_2622_MOESM3_ESM.docx]

**Rehabilitation guidelines for patients with Patella Dislocation**

**Phase 1 : 0-2 weeks**

- Extension splint
- Cryotherapy
- VMO activation with progression to supine Straight Leg Raise as pain allows
- Hip strengthening (abductors / adductors)
- Ankle pumps, isotonic exercises with Theraband
- Isometric quads / gluts / hamstrings
- Core stability
- Upper body exercises
- Fully weight bear as able with walking aid(s)

(Brace 0 degrees for 1 week, 0-30 degrees for 2^nd^ week)

**Phase 2 : 2-4 weeks**

0-60 degrees flexion week 3

0-90 degrees flexion week 4

Fully weight bear with brace locked for ambulation

Remove brace for week 4 if good quads control

**Phase 2 : 2-4 weeks Physio**

- Cryotherapy
- Electrical stimulation for VMO if not activating
- Bike with high seat
- Active Range Of Movement (ROM) /Active-Assisted ROM / Passive ROM to 60 degrees
- Quads sets, SLR 4 ways, Terminal Knee Extension (TKE) against Theraband
- Closed Kinetic Chain (CKC) Knee extensions (leg press, mini-stepups/squats, partial lunges)
- Total leg strengthening
- Balance / proprioception
- Cardiovascular conditioning
- Core stability
- Mc Connell taping / Kinesio taping if appropriate

**Phase 3 : 4-6 weeks+**

- Progress to full ROM
- Normalize gait
- Bike with resistance
- Flexibility exercises, normalise soft tissue length & strength
- Total leg strengthening, leg press, step-ups
- Squats and lunges to 90
- Biofeedback
- Balance and proprioception
- Mc Connell taping / Kinesio taping
- Identify & treat biomechanical abnormalities and activity modification

**Phase 4 : 12 weeks+**

- Return to running when 75% strength (usually 8-12 weeks for sports specific)
- Gradual return to plyometric activities
- Sports specific rehab / return to sport rehab
